# Assessment of some cultural experimental methods to study the effects of antibiotics on microbial activities in a soil: An incubation study

**DOI:** 10.1371/journal.pone.0180663

**Published:** 2017-07-06

**Authors:** Ali Molaei, Amir Lakzian, Gholamhosain Haghnia, Alireza Astaraei, MirHassan Rasouli-Sadaghiani, Maria Teresa Ceccherini, Rahul Datta

**Affiliations:** 1 Department of Soil Science, Faculty of Agriculture, Ferdowsi University of Mashhad, Mashhad, Iran; 2 Department of Soil Science, Faculty of Agriculture, Urmia University, Urmia, Iran; 3 Department of Agrifood and Environmental Science, University of Florence, Florence, Italy; 4 Department of Geology and Soil Science, Faculty of Forestry and Wood Technology, Mendel University, Brno, Czech Republic; RMIT University, AUSTRALIA

## Abstract

Oxytetracycline (OTC) and sulfamethoxazole (SMX) are two of most widely used antibiotics in livestock and poultry industry. After consumption of antibiotics, a major portion of these compounds is excreted through the feces and urine of animals. Land application of antibiotic-treated animal wastes has caused increasing concern about their adverse effects on ecosystem health. In this regard, inconsistent results have been reported regarding the effects of antibiotics on soil microbial activities. This study was conducted based on the completely randomized design to the measure microbial biomass carbon, cumulative respiration and iron (III) reduction bioassays. Concentrations of OTC and SMX including 0, 1, 10, 25, 50, and 100 mg/kg were spiked in triplicate to a sandy loam soil and incubated for 21 days at 25°C. Results showed that the effects of OTC and SMX antibiotics on cumulative respiration and microbial biomass carbon were different. SMX antibiotic significantly affected soil microbial biomass carbon and cumulative respiration at different treatments compared to control with increasing incubation time. OTC antibiotic, on the other hand, negatively affected cumulative respiration compared to control treatment throughout the incubation period. Although OTC antibiotic positively affected microbial biomass carbon at day one of incubation, there was no clear trend in microbial biomass carbon between different treatments of this antibiotic after that time period. Nevertheless, sulfamethoxazole and oxytetracycline antibiotics had similar effects on iron (III) reduction such that they considerably affected iron (III) reduction at 1 and 10 mg/kg, and iron (III) reduction was completely inhibited at concentrations above 10 mg/kg. Hence, according to our results, microbial biomass carbon and cumulative respiration experiments are not able alone to exhibit the effect of antibiotics on soil microbial activity, but combination of these two experiments with iron (III) reduction test could well display the effects of sulfamethoxazole (SMX) and oxytetracycline (OTC) antibiotics on soil biochemical activities.

## Introduction

Application of antibiotics is increasingly widespread in the livestock industry to facilitate animal growth and production and for the prevention of infectious diseases. The majority of antibiotics are excreted through animal's feces and urine and disseminated into the soil and nearby environment which may result in inhibition of non-target soil microbial species due to bioactive properties of antibiotics [1]. The residual concentrations of antibiotics have been reported in the range of a few micrograms up to few grams per kilogram in the soil environment [2] and from a few to more than 200 milligrams per liter or kilogram in manures [3].

After dissemination in soil, antibiotics may either get stabilized on soil matrix or be degraded by photohydrolysis or may undergo biotransformation [4]. A large fraction of antibiotics in soil meet the fate of photohydrolysis or biotransformation, both of which result in loss of antibiotic activity [5]. However, stabilization on soil matrix provides more stability to antibiotics by preventing degradation and result in longer persistence of these compounds in soil. Thus, it may impose environmental risks and be a potential hazard to soil microbiota which in turn may affect soil and human health [6]. Chander et al. [7] found that tetracycline (TC) bonded to soil particles retained its antibiotic activity and inhibited the growth of *Escherichia coli*, and sensitive and resistant strains of *Salmonella* bacteria. However, resistant *Salmonella* is inherently resistant; it showed lesser decline in growth compared to sensitive *Salmonella* and *E*. *coli*.

Inconsistent results have been reported about the effects of antibiotics on soil microbial activities. For example, Liu et al. [5] showed that soil enzymatic activities were significantly inhibited with chlortetracycline (CTC) treatments, while community level physiological profiles (CLPP) determined using the BIOLOG EcoPlate^TM^ system were less affected. However, in another experiment, OTC stimulated the soil enzyme activity and microbial biomass carbon [8]. Some pharmaceutical antibiotics changed the composition of soil microbial communities [9]. Soil microbial activity displayed a suppression-recovery-stimulation trend in chlortetracycline and carbendazim treatments [10]. Soil contaminated with sulfachloropyridazine (SCP) has little effect on community level physiological profiles (CLPP), while an increase in pollution-induced community tolerance (PICT) was reported with an increase in sulfachloropyridazine concentration [11]. Thus, the diversified effects of antibiotics on soil microbial communities may depend on type of experimental protocol followed, different antibiotics used and complex composition of microbial communities in soils.

Routine tests used to assess the effects of pollutants such as pesticides and herbicides may not be suitable for evaluating the effects of antibiotics since pharmaceutical antibiotics have some properties (e.g., inherent stability in soils and specific mode of action) that are different from other chemicals [12]. In fact, there are no established methods for assessment of antibiotics as pollutants on soil microflora. Therefore, the validity of the methods used to assess the effects of antibiotics is still in question.

The sensitivity of iron (III) reduction test as a bioassay for organic pollutants has been established for sulfonamides and tetracyclines antibiotics [13], and sulfadimethoxine (SDM) and monensin chemicals [14] in previous studies. However, according to Zelles et al. [15], a bioassay is not able to assess all diverse reactions due to heterogeneous microbial communities, and each trial method has its own weaknesses. When using the iron (III) reduction experiment, preparation of soil slurry may affect the availability of antibiotics, and also the adaptation of microorganisms during the incubation time of 7 days cannot be completely ignored [16]. Therefore, use of a combination of several experimental methods is recommended to evaluate the effects of antibiotics on soil microflora.

To observe the effect of pesticides on soil microflora, Imfeld and Vuilleumier [17] proposed the combination of one culture-independent method such as phospholipid fatty acid analysis (PLFA) and complementary analytical measurements such as bacterial 16SrRNA genotyping by terminal-restriction fragment length polymorphisms (T-RFLP). Widenfalk et al. [18] by combining these two methods found that herbicides of captan (0.0013–1.3 mg/kg), glyphosate (0.15–150 mg/kg), isoproturon (0.0052–5.2 mg/kg) and primicard (0.0022–2.2 mg/kg) affected soil bacterial structure of freshwater sediments at low concentrations, which are related to environmental concentrations. In contrast, in the same study, no response was observed on overall microbial activity determined alone by [^14^C]leucine incorporation technique and microbial biomass determined by PLFA, respectively [17].

Thus, in this study, we used the combination of conventional tests for measuring overall microbial activity: soil microbial biomass carbon, cumulative respiration, and iron (III) reduction test for assessing sulfamethoxazole (SMX), sulfonamides group, and oxytetracycline (OTC), tetracyclines groups, on soil biochemical activities. Our objectives in this study were (1) to study the combination of conventional and specific experiments for assessing pharmaceutical antibiotics on soil biochemical activities and (2) to study specific effects of antibiotics on soil biochemical activities.

## Materials and methods

### Soil sampling

The soil samples have been provided from my personal agricultural land, located in the countryside of Urmia, Iran. Therefore, I am responsible person for its management and conservation. Soil sampling was randomly carried out from the depth of 0 to 20 cm of soil. Then, soil samples were passed through 2 mm sieve and stored at 4°C in the dark until use.

Soil texture, total organic carbon and water holding capacity (WHC) were determined using the hydrometer method [19], Walkley-Black procedure [20], and a pressure plate method [21], respectively. Soil pH was measured in a ratio of soil to 0.01 M CaCl_2_ solution (1:2 w/v) [22]. Properties of the soil are presented in Table 1.

**Table 1:** Physical and chemical characterization of the soil.

Clay%	Sand%	Silt%	pH	Total organic carbon%	Water holding capacity (WHC)%
18.42	52.35	29.23	7.56	0.95	20

### Chemicals

**Fig 1:**
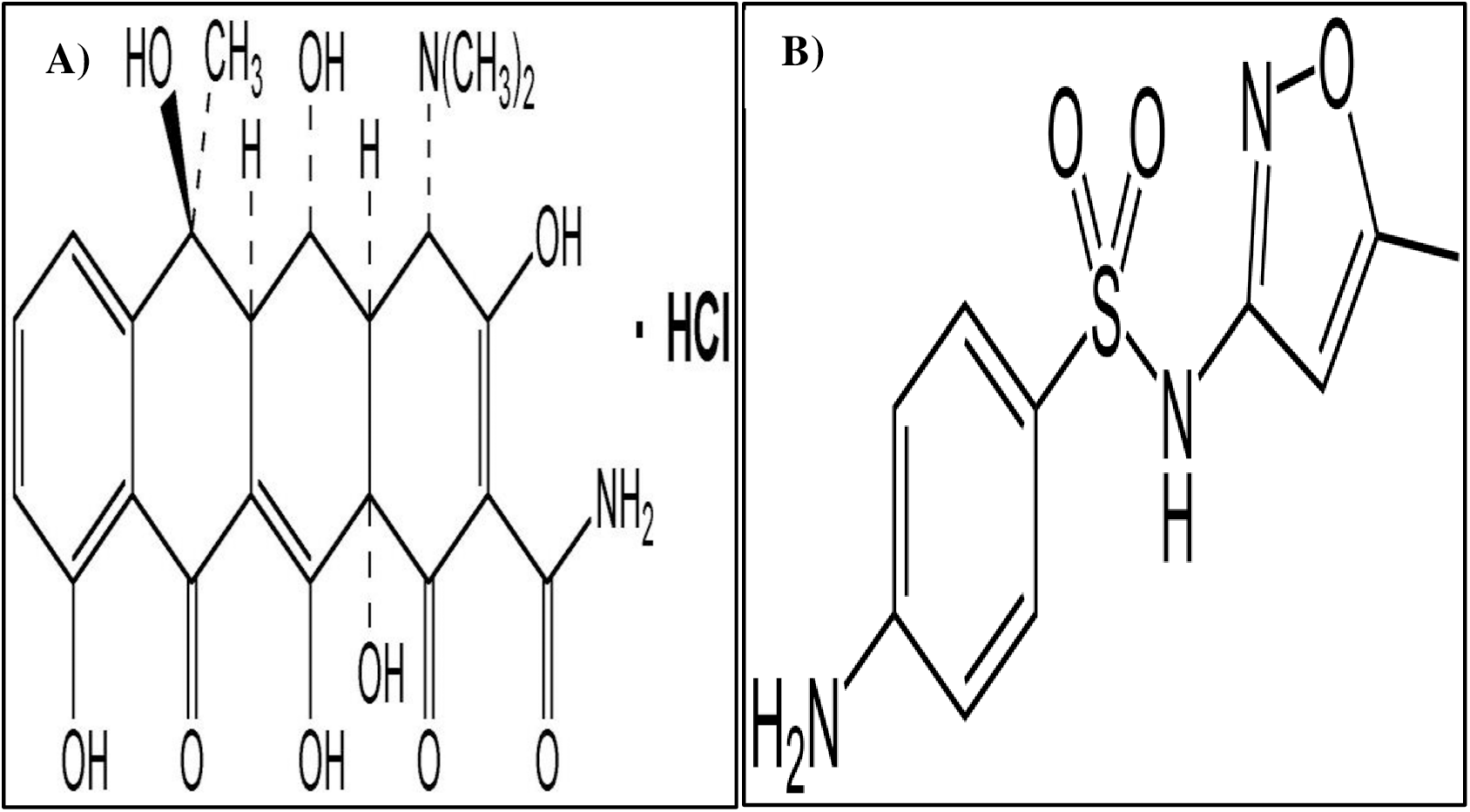
Chemical structures of oxytetracycline hydrochloride (A) and sulfamethoxazole (B).

Sulfamethoxazole (analytical standard, Italy, CAS 723-46-6) and oxytetracycline hydrochloride (≥95% (HPLC), China, CAS 2058-46-0) were purchased from Sigma-Aldrich. Chemical structures of these antibiotics are presented in Fig 1. All salts and acids were analytical grade reagents (Merck).

### Preparation of soil microcosms

To obtain a homogeneous distribution of antibiotics in the soil samples, the required values of sulfamethoxazole and oxytetracycline hydrochloride antibiotics [0 (control); 1 (normal); 10 and 25 (high); and 50 and 100 mg/kg (extremely high) levels] [23] accompanied with 2 g glucose per kg soil as a substrate were premixed with 10 g heated soil (600°C, 48 h) [24]. Afterward, these 10 g soils spiked with antibiotics were added to 200 g soil (dry weight equivalent). Finally, these soil samples were placed in 500 ml plastic trays with caps containing many small holes to allow gas exchange and minimize water evaporation. All soil microcosms were incubated at 25°C in the dark to avoid quick degradation of antibiotics and at optimum moisture content (50% of the field water capacity) for 21 days.

### Soil microbial biomass carbon and cumulative respiration

Soil microbial biomass carbon was determined by chloroform fumigation-extraction method [25]. For this purpose, soil samples were fumigated with chloroform for 24 h, extracted with potassium sulfate solution, and organic carbon was determined in the resulting extracts. Microbial biomass carbon was calculated using a K_EC_ of 0.35 [26].

Soil microbial cumulative respiration was determined by titration method [27]. For this purpose, soil samples were placed in the sealed containers including sodium hydroxide (NaOH) solution and incubated at 25°C. The remained NaOH was titrated by hydrochloric acid at different time intervals. The microbial respiration was expressed as a rate of mg CO_2_. g^-1^ dm. h^-1^.

### Iron (III) reduction test

Ten milliliters of ultra-pure water were added to the 10 g soil samples pre-spiked with antibiotics at concentrations of 0, 1, 10, 25, 50, and 100 mg/kg; the tubes were stoppered, incubated at 20°C for 48 h in the dark and agitated on a vertical rotary shaker to reach distribution equilibrium of the antibiotics and to recover microbial activity. After that period, microbial activity was facilitated by the addition of 150 mg glucose as nutrient substrate. The samples were incubated for 5 days at 20°C in the dark, agitated for 15 min every day beginning from day 2. After incubation, 10 ml 1 M KCl was added, and samples were vortexed for 1 min and centrifuged for 30 min at 5000 g. The supernatant was decanted quickly through a paper filter (filter 5951/2, Schleicher and Schuell, Dassel, Germany) and stabilized by the addition of 125 μl concentrated HNO3. The iron concentration of the solutions, indicative of the soil microbial activity, was analyzed using inductively coupled plasma-optical emission spectroscopy (Model 76004553/ CRMA, Spectro Arcos, Germany) at 260 nm and external standards [13].

### Data analysis

Statistical analyses were performed using the SAS, 9.2 software. Microbial parameter data were analyzed using a two-way analysis of variance (ANOVA) with antibiotic concentration and incubation time as factors. Differences between means for a given antibiotic concentration and incubation time were compared using a one-way ANOVA and Fisher's LSD post-hoc test. The values were considered to be significantly different at a 95% confidence level. The values in the figures correspond to the average of triplicate data ± standard deviations (SD).

## Results and discussion

### Soil microbial biomass carbon

Based on the results of analysis of variance (ANOVA), OTC and SMX antibiotics had significant effects on soil microbial biomass carbon at incubation period (Table 2). Besides, the highest changes in this microbial parameter were related to incubation time.

**Table 2:** Two-way analysis of variance (ANOVA) of microbial biomass carbon for OTC and SMX treatments.

Factor		OTC			SMX	
	Degree of Freedom	Sum of Square	F value	Degree of Freedom	Sum of Square	F value
**Time**	2	197287.68	2877.07***	2	63635.37	1838.55***
**Treatment**	5	29781.31	173.72***	5	11419.47	131.97***
Time^*^ **treatment**	10	58121.26	169.52***	10	35249.30	203.68***
**Error**	36	1234.30		36	623.01	
**Coefficient Variance**		6.83			5.42	

The categorical factors are incubation time (1, 4, and 21 days) and treatment (spiking concentrations: 0, 1, 10, 25, 50, and 100 mg/kg). F-values are presented with the level of significance (***, p˂0.001).

**Fig 2:**
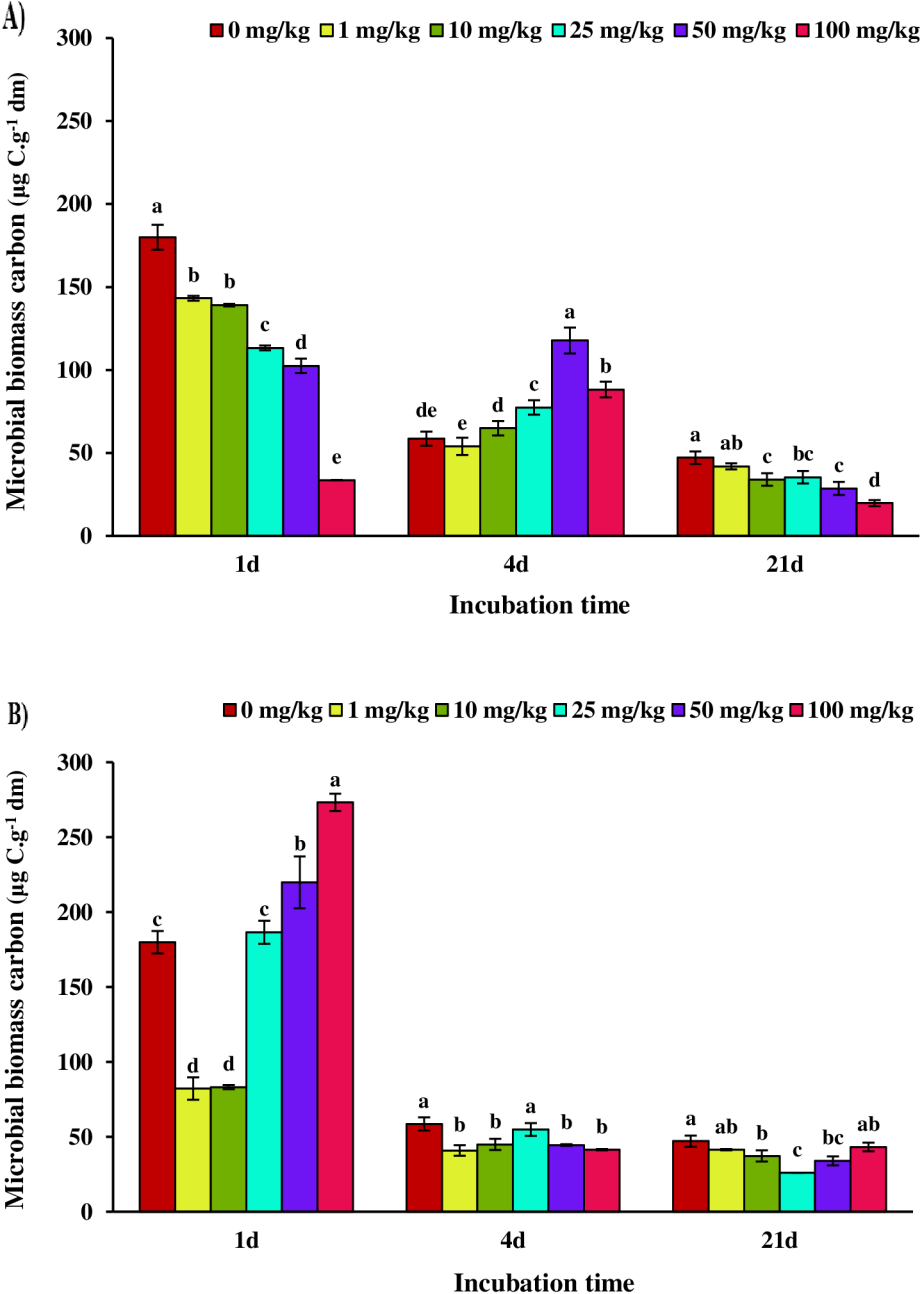
**Microbial biomass carbon of sulfamethoxazole (SMX) (A) and oxytetracycline (OTC) (B) treatments depending on sample treatment (0, 1, 10, 25, 50, and 100 mg/kg) and incubation time (1, 4 and 21 days).** Different letters indicate significant differences (confidence level of 95%) among treatments at a given incubation time (one-way ANOVA, followed by the least significant difference test). Error bars represent the standard deviation of replicate analyses (n = 3).

After addition of SMX to soil samples for 24 hours, we observed that soil microbial biomass carbon was significantly (p≤0.05) reduced with increasing concentrations of SMX (Fig 2A). The reduction in microbial biomass carbon of 1, 10, 25, 50, and 100 mg/kg treatments were 20%, 22%, 37%, 43%, and 81% respectively with respect to control. Thus, the highest inhibition of soil microbial activity was at the highest SMX concentration indicating toxicity of SMX antibiotic on soil microbial community. In contrast, with increasing the time of incubation to 4d, soil microbial biomass carbon of 25, 50, and 100 mg/kg treatments significantly (p≤0.05) increased with respect to control, and 1 and 10 mg/kg treatments. Different studies have shown that bacteriostatic antibiotics such as SMX have adverse effects on soil bacteria, while their adverse effects have not been observed on fungal activity [28]. For example, Thiele-Bruhn and Beck [24] observed that sulfapyridine (SPY) antibiotic had no effect on ergosterol concentration as an indicator of fungal biomass. In another study, the soils treated with pig manure containing SDZ exhibited the shifts in the microbial community with an increase in fungi and a decrease in bacteria population as shown by PLFA profiles in the rhizosphere and bulk soils [29]. Therefore, one of the reasons for the increase in soil microbial biomass carbon at higher concentrations of SMX might be the higher fungal biomass due to suppression of soil bacterial activity by this antibiotic, which leads to an increase in the bioavailable substrate for growth of soil fungi; termed as cryptic growth [30]. Interestingly, by increasing incubation time to 21 days, the adverse effects of SMX on soil microbial biomass carbon was similar to that observed for 24 h incubation treatments; soil microbial biomass carbon was significantly (p≤0.05) reduced with increasing concentration of SMX antibiotic. However, it was expected that due to the reduction in bioavailability of antibiotics over time, their impact on soil microbial community may be reduced [31]. According to Thiele-Bruhn and Beck [24], SPY antibiotic was undetectable in the soil samples during 14 days of incubation. However, the effect of this antibiotic on soil microflora was still visible. Westergaard et al. [32] also observed the changes in microbial community composition in tylosin contaminated soils after 2 months of incubation. Furthermore, inhibitory effects of metabolites resulting from decomposition of SDZ such as OH-SDZ-4 and N-Ac-SDZ have been reported in pig manure treated with SDZ [29]. The similar adverse effects of metabolites have also been observed for many other antibiotics such as tetracyclines (TCs) [33]. In fact, it can be indicated from these findings that the changes in soil microbial biomass carbon may continue for a longer time, up to a few weeks, due to SMX contamination.

The effect of OTC antibiotic on soil microbial biomass carbon was different from that of SMX antibiotic (Fig 2). Given that the bioavailability of antibiotics is highest on the first day of incubation, the highest inhibitory effect is expected on soil microflora especially soil bacteria. However, as shown in Fig 2B, OTC antibiotic resulted in an increase in microbial biomass carbon at high concentrations (50 and 100 mg/kg treatments) in comparison with control treatment on the first day of incubation. Liu et al. [5] showed that soil microbiota could use tetracyclines antibiotics as a carbon source and cause an increase in soil microbial activity. With increasing incubation time to 4 days, soil microbial biomass carbon significantly (p≤0.05) decreased at different OTC concentrations when compared to control except for 25 mg/kg treatment. Although, it has been already reported that OTC can significantly reduce soil microbial biomass [34], on day 21 of incubation, no significant difference (p≤0.05) was observed between 100 mg/kg treatment and control. The high potential of tetracyclines antibiotics for rapid absorption onto the soil matrix have been determined [35] such that Liu et al. [5] found that extractable concentrations of chlortetracycline antibiotic rapidly decreased after soil treatment with this antibiotic. However, in the present study, the effect of OTC antibiotic on soil microbial biomass carbon did not follow a clear trend for different treatments at all incubation period.

### Soil microbial cumulative respiration

Soil microbial respiration was measured as an indicator of soil microbial activities for assessment effects of SMX and OTC antibiotics in the soil treatments. Based on the ANOVA results, there were significance differences in cumulative respiration at different treatments of OTC and SMX antibiotics during incubation (Table 3).

**Table 3:** Two-way analysis of variance (ANOVA) of cumulative respiration for OTC and SMX treatments.

Factor		OTC			SMX	
	Degree of Freedom	Sum of Square	F value	Degree of Freedom	Sum of Square	F value
**Time**	7	95.59	13506.9***	7	103.72	6791.3***
**Treatment**	5	2.17	430.31***	5	0.52	48.29***
Time^*^ **treatment**	35	0.35	10.16***	35	2.15	28.26***
**Error**	96	0.097		96	0.209	
**Coefficient Variance**		1.49			2.09	

The categorical factors are incubation time (1, 2, 3, 4, 5, 6, 11, and 21 days) and treatment (spiking concentrations: 0, 1, 10, 25, 50, and 100 mg/kg). F-values are presented with the level of significance (***, p˂0.001).

**Fig 3:**
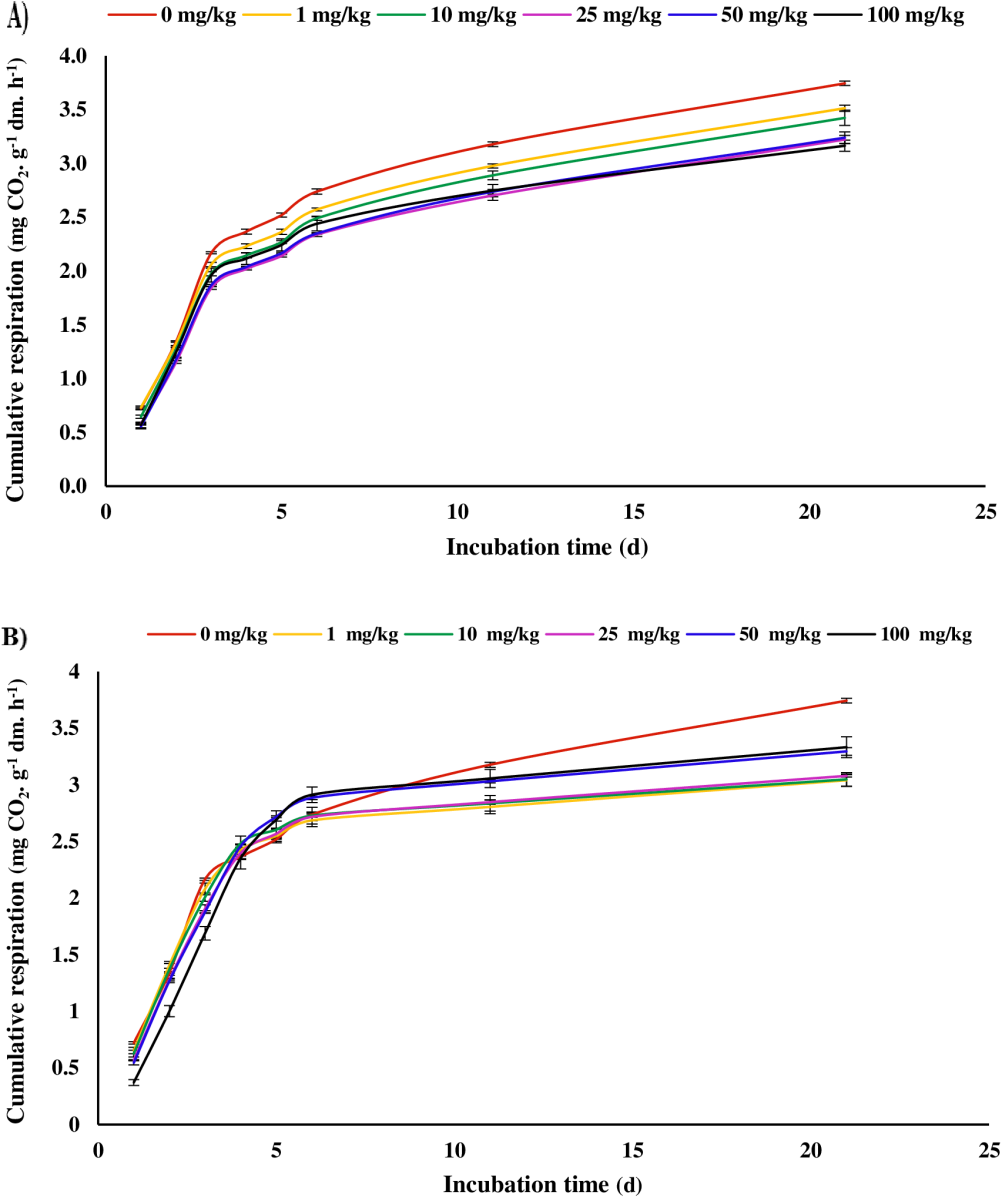
**Cumulative respiration of oxytetracycline (OTC) (A) and sulfamethoxazole (SMX) (B) treatments depending on sample treatment (0, 1, 10, 25, 50, and 100 mg/kg) and incubation time (1, 2, 3, 4, 5, 6, 11, and 21 days).** Error bars represent the standard deviation of replicate analyses (n = 3). The standard deviations of replicates are in the range of 0.005–0.071 and 0.006–0.095 mg CO_2_. g^-1^ dm. h^-1^ for oxytetracycline and sulfamethoxazole treatments, respectively.

The cumulative respiration of soil microflora in soil samples contaminated with OTC antibiotic is shown in Fig 3A. No significant differences (p≤0.05) were observed in soil microbial respiration between different treatments of OTC antibiotic and control treatment for 6-hours incubation (data not shown). This is not surprising result, because the antimicrobial activity of OTC is based on the inhibition of protein synthesis, and thus this antibiotic should essentially inhibit microbial growth [36]. To observe the effects of bacteriostatic antibiotics such as OTC, incubation period should be long enough to provide optimum condition for microbial growth. Accordingly, in our experiment the inhibition of respiratory activity was observed for 24 hours of incubation (Fig 3A). The cumulative respiration was 0.721 (± 0.011) mg CO_2_. g^-1^ dm. h^-1^ for the control, and 0.577 (± 0.009), 0.554 (± 0.022), and 0.568 (± 0.026) mg CO_2_. g^-1^ dm. h^-1^ for 25, 50, and 100 mg/kg treatments, respectively. The cumulative respiration strongly increased in all antibiotic treatments leading up to the third day of incubation. However, the difference in cumulative respiration between OTC treatments (2.059 ± 0.020, 1.974 ± 0.019, 1.841 ± 0.013, 1.867 ± 0.008, and 1.961 ± 0.064 mg CO_2_. g^-1^ dm. h^-1^ for 1, 10, 25, 50, and 100 mg/kg treatments, respectively) and control (2.167 ± 0.009 mg CO_2_. g^-1^ dm. h^-1^) was significant (p≤0.05) on 3 days of incubation and the highest cumulative microbial respiration was observed in control treatment. Upon extending the incubation time to 11 days, cumulative microbial respiration increased gently. Besides, the cumulative respiration kinetics showed that the inhibition of microbial activity also increased by increasing OTC concentrations. On the other side, cumulative microbial respiration significantly (p≤0.05) decreased in 100 mg OTC per kg soil than other treatments including the control on 21 days of incubation. These results showed that OTC had inhibitory effects on soil microbial activity. Assuming that some part of applied OTC became stabilized after 21 d in soil, residual concentrations of antibiotic are still high enough to exert some effects on soil microbial activity, or growth of soil microflora may have been still under the influence of OTC applied on the soil in the beginning [36]. Reichel et al. [29] observed the antimicrobial activity of residual SDZ and DFX antibiotics despite the gradual reduction of these compounds in 63 days’ incubation. Although the residual concentrations of antibiotics are not immediately bioavailable, they act as a long-term source for the release of antibiotics.

A significant reduction (p≤0.05) was observed in microbial cumulative respiration at concentration of 100 mg SMX per kg soil (ex: 0.372 ± 0.026 mg CO_2_. g^-1^ dm. h^-1^ on 1 day) than all of the rest concentrations including the control treatment (ex: 0.721 ± 0.011 mg CO_2_. g^-1^ dm. h^-1^ on 1 day) during 3 days of incubation (Fig 3B). These findings indicated the high impact of SMX antibiotic on soil microbial community [37]. Due to low distribution coefficient (Kd) value of SMX and therefore the low absorption of this antibiotic to soil particles, the stronger effect of SMX on soil microbial activity was a reasonable result [38]. However, there was no significant difference (p≤0.05) in cumulative respiration at different treatments of SMX antibiotic with respect to control on days of 4, 5 and 6. The main processes reducing the antimicrobial activity of antibiotics after being added to soil include inactivation of antibiotic compounds by chemical or biological changes, chelation with polyvalent cations, biodegradation, and absorption by soil particles [39]. Besides, since sulfonamides biodegradation rarely occurs in soils [40], strong absorption and consequently a decrease in bioavailability of antibiotics could to some extent explain the lack of significant difference of cumulative respiration between SMX treatments and control on 4, 5 and 6 days of incubation. With increasing incubation time up to 21 days, the cumulative respiration was at a higher level in control treatment in comparison with other treatments. Hammesfahr et al. [41] found that antibacterial effects of sulfonamides were increased during the weeks of incubation despite the reduction in bioavailable portions of antibiotics to concentrations below the limit of detection. Reichel et al. [29] reported that continuous remobilization of residual antibiotics by soil microflora associated with the same sorptive soil surfaces is a reason for the increase in antibiotic effects on soil microorganisms. Therefore, microorganisms bounded to soil matrix may absorb remobilized antibiotic compounds and be affected by their adverse effects.

### Iron (III) reduction test

**Fig 4:**
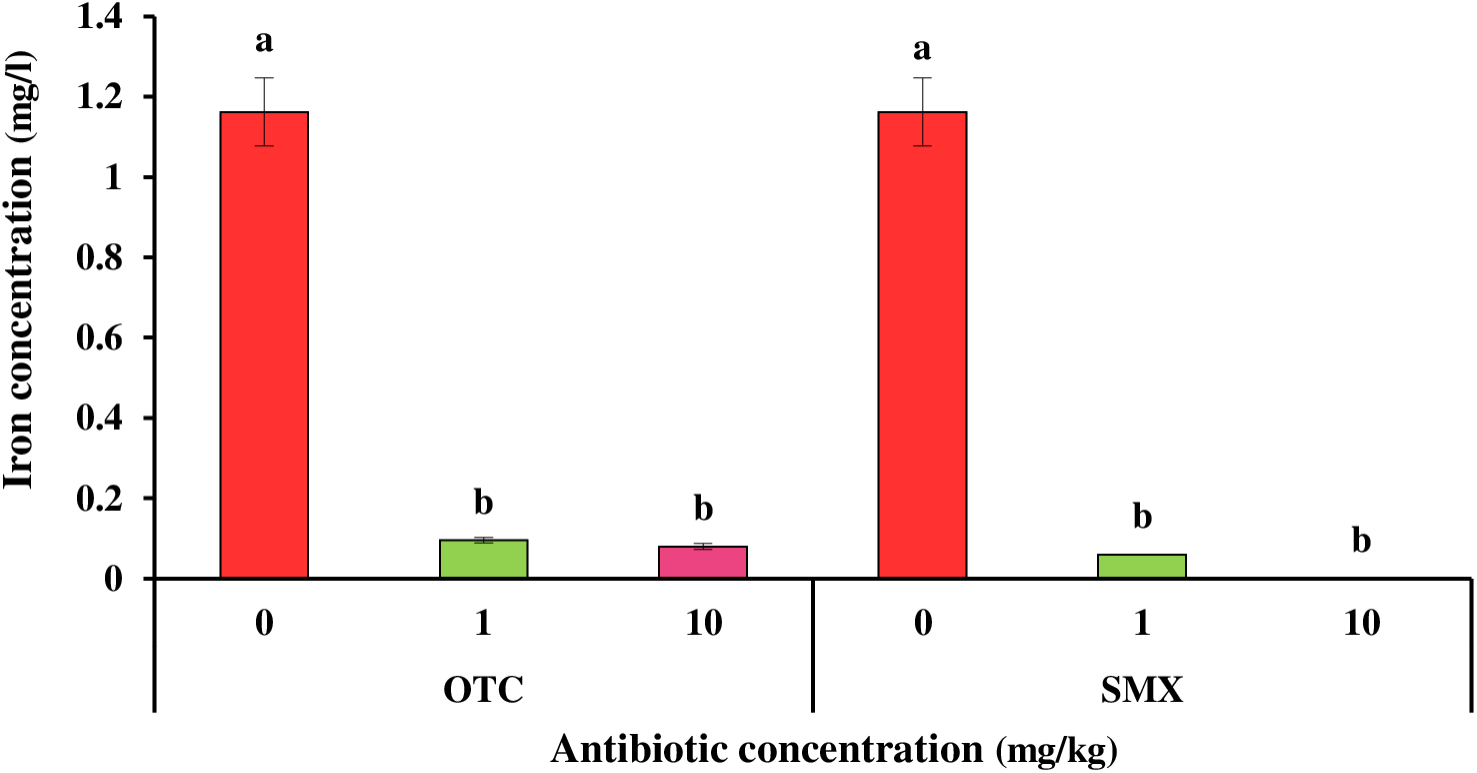
Iron concentration of oxytetracycline (OTC) and sulfamethoxazole (SMX) treatments depending on sample treatment (0, 1 and 10 mg/kg). Different letters indicate significant differences (confidence level of 95%) among treatments at incubation time (one-way ANOVA, followed by the least significant difference test). Error bars represent the standard deviation of replicate analyses (n = 3).

Different concentrations of OTC and SMX antibiotics including 1, 10, 25, 50, and 100 mg/kg influenced the reduction of iron from ferric to ferrous state. The effects of these antibiotics depend on the concentrations of the used compounds, as concentrations-response relationships obtained between various concentrations of antibiotics and iron (III) reduction. With increasing concentration of antibiotics, there was a sharp decrease in the iron (III) reduction (Fig 4). The clear dose-response relationships observed between different concentrations of OTC and SMX antibiotics and control treatment might be attributed to severe antibiotics effects of these compounds on soil microbial activities [42]. The iron concentration in treatments of 1 and 10 mg/kg decreased 91 and 93 percent for OTC antibiotic, and 94 and 99 percent for SMX antibiotic as compared to the control treatment, respectively (Fig 4). In line with our results, Reichel et al. [29] observed a significant increase in the amount of qCO_2_ at 1 mg SDZ kg soil that reflects the high tension response of soil microbial communities to antibiotics at environmental related concentrations. The remarkable point is that iron concentration at 25, 50 and 100 mg/kg treatments was below the detection limit of inductively coupled plasma-optical emission spectroscopy (data not shown). In fact, high antibiotic concentrations led to the complete removal of soil microbial iron (III) reduction. These findings indicated that both antibiotics had toxic effects on soil bacteria at concentrations relevant to the environment (low concentrations), and had a direct inhibitory effect on the soil bacteria growth as shown by Toth et al. [14]. These results confirmed that iron (III) reduction test is a suitable method to explain the effect of antibiotics on soil bacteria. Contrary to complete inhibition of microbial activity at higher concentrations of SMX and OTC in iron (III) reduction test (Fig 4), fewer adverse effects or even growth improvements were observed in soil microbial biomass carbon and cumulative respiration at soil samples contaminated with these antibiotics (Figs 2 and 3). Consistent with our results, Zelles et al. [15] found that despite a complete inhibition of microbial activity determined by iron (III) reduction test, no effects on soil fungus or even growth improvement were detected in soil samples treated with antibiotics. In fact, complete concentration-response relations obtained might be somewhat specific for iron (III) reduction test.

In addition, both OTC and SMX antibiotics had similar effects on iron (III) reduction, but the effect of SMX was slightly more than that of OTC. Contrary to our results, Thiele-Bruhn [13] showed that despite the overlap of both antibiotics in ED50 quantities, the toxicity of tetracyclines was slightly more than that of sulfonamides: the minimum inhibitory concentration (MIC) for cultivated microorganisms have been reported 0.5 to 2 mg/l and 0.1 to 64 mg/l for tetracyclines and sulfonamides, respectively [13]. This discrepancy may be due to different capabilities of soil microorganisms to counter antibiotics.

Although iron (III) reduction test has been designed in such way to reflect soil microbial activities involved in Fe reduction, soil fungi activity is limited in the first stage of oxygen consumption, and in the second anaerobic stage, anaerobic soil bacteria contribute to the reduction of iron (III) [43]. In this study, soil microbial activity was still retained at all concentrations of OTC and SMX treatments in soil microbial biomass carbon and cumulative respiration experiments. In comparison, soil microbial activity was completely inhibited at concentrations above 10 mg/kg in iron (III) experiment. Therefore, the present results provided evidence that oxytetracycline (OTC) and sulfamethoxazole (SMX) antibiotics more effectively inhibited soil bacterial activity.

## Conclusions

Microbial biomass carbon, cumulative respiration, and iron (III) reduction tests were examined on soil treated with increasing concentrations of oxytetracycline (OTC) and sulfamethoxazole (SMX) antibiotics. The results of the present study showed that effect of OTC and SMX on cumulative respiration and microbial biomass carbon followed a different pattern. Microbial biomass carbon and cumulative respiration were decreased with increasing concentration of sulfamethoxazole. In comparison, oxytetracycline antibiotic had no adverse effects on microbial biomass carbon. However, the cumulative respiration was decreased at different concentrations of OTC with respect to the control during the incubation. Therefore, we concluded that microbial biomass carbon and cumulative respiration parameters are not sufficient alone to exhibit the effects of oxytetracycline antibiotic on soil microbial activity; rather, these parameters can display effects of sulfamethoxazole antibiotic on soil microbial activity. However, the iron (III) reduction test well presented the effects of both antibiotics on soil biochemical activities. Therefore, the combination of microbial biomass carbon, cumulative respiration and iron (III) reduction bioassays could well display the effects of sulfamethoxazole (SMX) and oxytetracycline (OTC) antibiotics on soil biochemical activities.
